# Exploring the nanomechanical concepts of development through recent updates in magnetically guided system

**DOI:** 10.1038/s41598-021-92440-4

**Published:** 2021-06-30

**Authors:** Noor Saeed Khan, Auwalu Hamisu Usman, Attapol Kaewkhao, Poom Kumam, Phatiphat Thounthong, Usa Wannasingha Humphries

**Affiliations:** 1 KMUTTFixed Point Research Laboratory, Room SCL 802 Fixed Point Laboratory, Science Laboratory Building, Department of Mathematics, Faculty of Science, King Mongkut’s University of Technology Thonburi (KMUTT), Bangkok, 10140 Thailand; 2 Center of Excellence in Theoretical and Computational Science (TaCS-CoE), Science Laboratory Building, Faculty of Science, King Mongkut’s University of Technology Thonburi (KMUTT), 126 Pracha-Uthit Road, Bang Mod, Thrung Khru, Bangkok, 10140 Thailand; 3 Department of Medical Research, China Medical University Hospital, China Medical University, Taichung, 40402 Taiwan; 4 Division of Science and Technology, Department of Mathematics, University of Education Lahore, Lahore, 54770 Pakistan; 5 Department of Mathematical Sciences, Faculty of Physical Sciences, Bayero University Kano, Kano, Nigeria; 6 Data Science Research Center, Department of Mathematics, Faculty of Science, Chiang Mai University, Chiang Mai, 50200 Thailand; 7 Department of Teacher Training in Electrical Engineering, Renewable Energy Research Centre, Faculty of Technical Education, King Mongkut’s University of Technology North Bangkok, 1518 Pracharat 1 Road, Bangsue, Bangkok, 10800 Thailand

**Keywords:** Engineering, Materials science, Mathematics and computing, Nanoscience and technology, Physics

## Abstract

This article outlines an analytical analysis of unsteady mixed bioconvection buoyancy-driven nanofluid thermodynamics and gyrotactic microorganisms motion in the stagnation domain of the impulsively rotating sphere with convective boundary conditions. To make the equations physically realistic, zero mass transfer boundary conditions have been used. The Brownian motion and thermophoresis effects are incorporated in the nanofluid model. Magnetic dipole effect has been implemented. A system of partial differential equations is used to represent thermodynamics and gyrotactic microorganisms motion, which is then transformed into dimensionless ordinary differential equations. The solution methodology is involved by homotopy analysis method. The results obtained are based on the effect of dimensionless parameters on the velocity, temperature, nanoparticles concentration and density of the motile microorganisms profiles. The primary velocity increases as the mixed convection and viscoelastic parameters are increased while it decreases as the buoyancy ratio, ferro-hydrodynamic interaction and rotation parameters are increased. The secondary velocity decreases as viscoelastic parameter increases while it increases as the rotation parameter increases. Temperature is reduced as the Prandtl number and thermophoresis parameter are increased. The nanoparticles concentration is increased as the Brownian motion parameter increases. The motile density of gyrotactic microorganisms increases as the bioconvection Rayleigh number, rotation parameter and thermal Biot number are increased.

## Introduction

In the presence of an external magnetic field, ferrofluids (a portmanteau of liquid and ferromagnetic particles) are magnetized liquid. These fluids are colloidal liquids made up of nanoscale ferrimagnetic or ferromagnetic particles that are suspended inside a fluid transport mechanism (usually a water or organic solvent). Brownian motion causes particle suspension, but these particles do not settle under normal conditions. Furthermore, each ferromagnetic particle is coated with a surfactant to prevent clumping. Magnetic attraction of nanoscale ferromagnetic particles is weak when the van der Waals force of the surfactant is strong enough to prevent agglomeration or magnetic clumping. Ferromagnetic fluids have a wide range of applications like friction-reducing agent, angular momentum change agent, heat transfer agent, and other applications including electronic devices, analytical instruments, and medicine^1–3^. Due to these numerous applications, many scientists and researchers have accelerated the study of ferrofluid. Andersson and Vanes^4^ investigated the effect of a magnetic dipole on ferrofluid. Zeeshan et al.^5^ reported the mixed convection flow and heat transfer in ferromagnetic fluid over a stretching sheet with partial slip effects. Hayat et al.^6^ investigated the effects of thermal radiation and magnetic dipole on the flow of ferromagnetic Williamson liquid past a stretched surface. Some MHD studies can be read from the references^7–17^.

Rotating flows and heat transfer levels over stationary spinning bodies with a revolution in forced flow are important in a wide range of engineering applications like missile re-entry, projectile motion, fiber coating, and rotating machine design. Many researchers have already studied its axis of revolution parallel to the free flow velocity to solve problems of heat transfer and fluid flow on a rotating sphere. Here are some studies on this topic^18–28^. However, most recently, Mahdy^29^ investigated the concurrent effects of MHD and varying wall temperatures on the transient mixed Casson nanofluid flow at the stagnation point of the rotating sphere. Mahdy and Hossam^30^ reported the time-mixed convective nanofluid flow from the stagnaion point area of the fast rotating sphere of Newtonian’s heating with microorganisms. Some other rotating flows investigations exist in the references^31–33^.

In perspective of the pervasive engineering applications and industry, the study of non-Newtonian fluid flows has indeed achieved an exemplary commitment. Materials exhibiting a non-linear relationship between strain rate and stress, with variable coefficient viscosity, and belongings described in the Navier–Stokes equation, are recognized as non-Newtonian fluids. Many industrial fluid processes, like molten polymers, paper production, paints, cosmetics, clay mixtures, oil recovery, food dispensation and movement of biological fluids, exhibit non-Newtonian characteristics. Several models of non-Newtonian material have been developed. However, in general, the flow equations are often more non-linear compared to Newtonian fluid equations. For these reasons, the research area of non-Newtonian fluids brings some exciting and interesting difficulties for engineers, mathematicians, and computer scientists alike. Ibrahim^34^ provided the numerical analysis of time-dependent flow of viscous fluid due to a stretchable rotating disk with heat and mass transfer. Ibrahim et al.^35^ studied the numerical simulation and sensitivity analysis of a non-Newtonian fluid flow inside a square chamber exposed to a magnetic field using the FDLBM approach. Interesting recent studies on non-Newtonian fluids have been conducted by various researchers^36–41^. The second-grade fluid model is the kindest subtype of viscoelastic fluid that can be expected for analytical solution as in^42–45^.

A larger percentage of nuclear and thermal-hydraulic systems requires the heat transmissions that attempt to incorporate fluid flow. A wide range of fluids and operating conditions have been verified to enhance the heat transport process. The interaction of such fluids with the existing system can help to reduce capital costs, improve working efficiency, and the design of the system. Furthermore, the involvement of cooling in several technological processes, such as engines, laptops, computers, electrical strips, is crucial to maintain the effective thermal performance of these products. Nanofluid is the attribute of a nanometer-sized particle suspended in liquid. Nanofluid has been shown to improve the thermal conductivity of the base fluid under the influence of such suspendend particles, and this concept originates from the work of Choi and Eastman^46^, which is then clarified by several researchers. El-Shorbagy et al.^47^ investigated numerically the mixed convection in nanofluid flow in a trapezoidal channel with different aspect ratios in the presence of porous medium. Ali et al.^48^ examined the navigation effect of tungsten oxide nano-powder on ethylene glycol surface tension by artificial neural network and response surface methodology. Chu et al.^49^ studied the rheological behavior of MWCNT-TiO2/5W40 hybrid nanofluid based on experiments and RSM/ANN modeling. This leads to a number of researchers to see the huge efficient flow of nanofluids under different aspects^50,51,53–57^.

Bioconvection is a new type of industrial and biological fluid mechanics in which the phenomenon of it arises from macroscopic convective fluid movements due to the inclusion of upwardly swimming motile microorganisms (denser than the medium). As a result, the agglomerate of the loaded self-propelled microorganisms on the liquid upper surface occurs, where it contributes to density stratification, is unstable and results in the image of the bioconvection dust clouds. Water must be a standard fluid so that these self-propelled microorganisms are active in the base fluid^58–66^.

Inspired mostly by the above-mentioned references to bioconvection nanofluid flows, the purpose of this paper is to examine the behavior of transient mixed stagnation point boundary layer flow of second-grade fluid across the rashly revolving sphere consisting of nanoparticles and motile gyrotactic microorganisms under magnetic dipole effects. The impetuous movement of nanofluid, microorganisms and the impulsive rotation of the sphere lead to unsteadiness. Similarity transformation approach is used to determine the non-linear ordinary differential equations and solution through the homotoy analysis method HAM^67–70^.

## Problem formulation

The two-dimensional time-state, laminar incompressible, mixed convection boundary layer flow of viscous electrically conducting second grade nanofluid with swimming gyrotactic microorganisms in the stagnation domain of the rotating sphere and thermal convective boundary condition is scrutinized. Recognizing the Buongiorno nanofluid model, comprising of Brownian motion and thermophoresis effects is adopted in the present investigations. It is assumed that uniform dispersion is achieved by the lack of aggregation and accretion of nanoparticles. The sphere is revolving with a constant angular velocity $\Omega$. At time *t* = 0, the sphere is at rest and the surface temperature, concentration and motile microorganisms are $T_{\infty }$, $C_{\infty }$ and $N_{\infty }$ in an ambient fluid, respectively. The flow of motile microorganisms remains constant along sphere surface and zero mass flux condition is applied at the surface. The sphere surface is warmed due to convection from a warm nanofluid at the temperature $T_{\infty }$ which provides a heat transfer factor ${h}_{{f}}$, is to be strengthen or weaken to the value $T_{\infty }$, where $T_w > T_{\infty }$ leads to aiding flow and $T_w < T_{\infty }$ leads to reversing flow, respectively. Apart from nanofluid properties, which are chosen to be constant, density variation is based on Boussinesq approximation. Both Joule heating and viscous dissipation effects have been ignored. Nanoparticles have no influence on the direction and velocity of gyrotactic microorganisms. Motile microorganisms, nanoparticles and base fluid have the same velocity.

**Figure 1:**
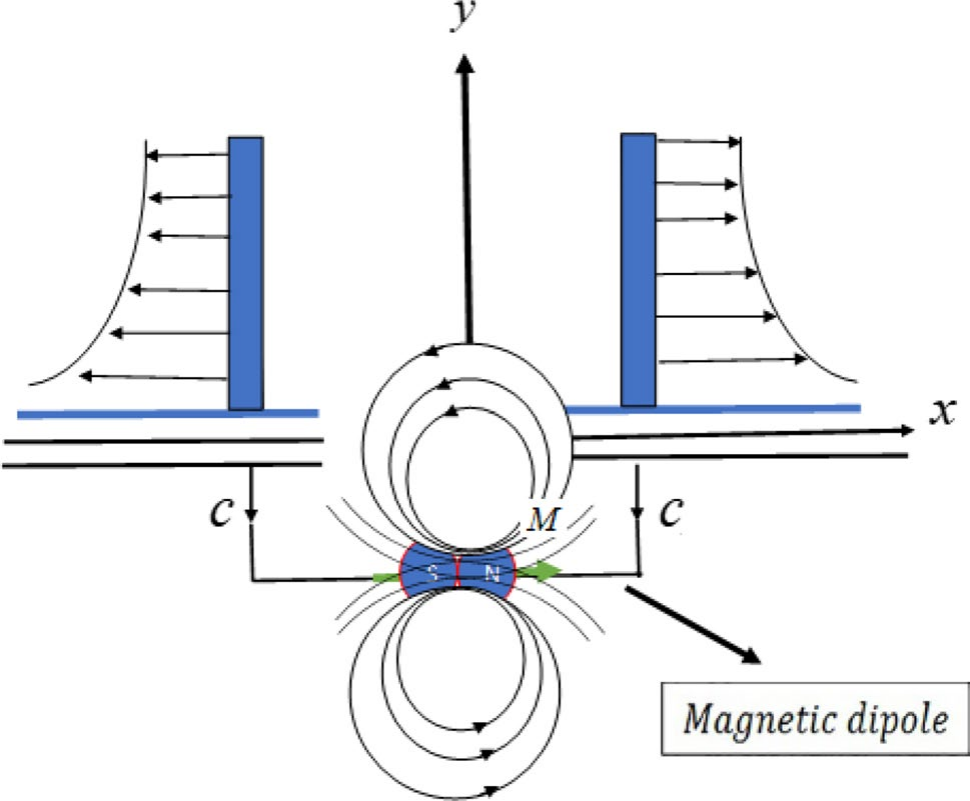
Schematic diagram of the problem.

### Magnetic dipole

The characteristics of the magnetic field have an effect on the flow of ferrofluid due to the magnetic dipole^2,4,6^. Magnetic dipole effects are recognized by the magnetic scalar potential $\Phi$ shown as in Eq. (1)

1 $$\begin{aligned}&\Phi =\frac{\gamma }{2\pi }\frac{x}{x^2+(y+c)^2}, \end{aligned}$$

where $\gamma$ stands for the magnetic field strength at the source. Taking $H_x$ and $H_y$ as the components of magnetic field as shown in Eqs. (2) and (3),

2 $$\begin{aligned}&H_x=-\frac{\partial \Phi }{\partial x}=\frac{\gamma }{2\pi }\frac{x^2-(y+c)^2}{[x^2+(y+c)^2]^2}, \end{aligned}$$

3 $$\begin{aligned}&H_y=-\frac{\partial \Phi }{\partial y}=\frac{\gamma }{2\pi }\frac{2x(y+c)}{[x^2+(y+c)^2]^2}. \end{aligned}$$

Since the magnetic body strength is usually proportional to *H*_*x*_ and *H*_*y*_ gradient, *c* is the distance of the line currents from the leading edge, it is therefore given as in (4)

4 $$\begin{aligned} H=\sqrt{H_{x}^{2}+H_{y}^{2}}. \end{aligned}$$

Equation (5) can approximate the linear shape of magnetization *M* by temperature *T* as

5 $$\begin{aligned}&M=K_{1}(T-T_\infty ). \end{aligned}$$

The value of $K_1$ is identified as a ferromagnetic coefficient. Figure 1 shows the physical diagram of the problem about magnetic dipole.

The problem equations for second grade nanofluid with dipole effect are given as^21–23,29,30,43–45^

6 $$\begin{aligned}&\frac{\partial (xu)}{\partial x} + \frac{\partial (xv)}{\partial y} = 0, \end{aligned}$$

7 $$\begin{aligned}&\frac{\partial u}{\partial t}+u \frac{\partial u}{\partial x}+w \frac{\partial u}{\partial y}-\frac{v^2}{x} = U \frac{\partial U}{\partial x} +\nu _{f}\frac{\partial ^2 u}{\partial y^2} \\&\quad + \frac{\alpha _{1}}{\rho _{f_\infty }} \left[ u \frac{\partial ^{3}u}{\partial x\partial y^{2}} + v \frac{\partial ^{3}u}{\partial y^{3}} + \frac{\partial u}{\partial x} \frac{\partial ^{2}u}{\partial y^{2}} - \frac{\partial u}{\partial y} \frac{\partial ^{2}u}{\partial x\partial y} + \frac{\partial w}{\partial y} \frac{\partial ^{2}w}{\partial x\partial y} + \frac{\partial w}{\partial x} \frac{\partial ^{2}w}{\partial y^{2}} \right] +\frac{\mu _{o}M}{\rho _{f_\infty }}\frac{\partial H}{\partial x} \\&\quad +\left[(1-C_\infty )\beta \rho _{f_\infty }(T - T _{\infty } ) - (\rho _p-\rho _{f_\infty })(C - C _{\infty } )-(\rho _m-\rho _{f_\infty })\gamma ^{*}(N - N_{\infty })\right]\frac{gx}{R\rho _{f_\infty }}, \end{aligned}$$

8 $$\begin{aligned}&\frac{\partial v}{\partial t}+u \frac{\partial v}{\partial x}+w \frac{\partial v}{\partial y}-\frac{uv}{x}=\frac{\mu _{f_\infty }}{\rho _{f_\infty }}\frac{\partial ^2 v}{\partial y^2} + \frac{\alpha _{1}}{\rho _{f_\infty }} \left[ u \frac{\partial ^{3}w}{\partial x\partial y^{2}} + v \frac{\partial ^{3}w}{\partial y^{3}}\right], \end{aligned}$$

9 $$\begin{aligned}&(c_p\rho )_{f_\infty }\left( \frac{\partial T}{\partial t}+u \frac{\partial T}{\partial x}+w \frac{\partial T}{\partial y}\right) =k_f\frac{\partial ^2 T}{\partial y^2}+\tau \left( D_B\frac{\partial C}{\partial y}\frac{\partial T}{\partial y}+\frac{D_T}{T_\infty }\left( \frac{\partial T}{\partial y}\right) ^2\right) \nonumber \\&\quad + \bigg (u\frac{\partial H}{\partial x}+v\frac{\partial H}{\partial y}\bigg ){\mu _o}T\frac{\partial M}{\partial T}, \end{aligned}$$

10 $$\begin{aligned}&\frac{\partial C}{\partial t}+u \frac{\partial C}{\partial x}+w \frac{\partial C}{\partial y}=D_B\frac{\partial ^2 C}{\partial y^2}+\frac{D_T}{T_\infty }\frac{\partial ^2 T}{\partial y^2}, \end{aligned}$$

11 $$\begin{aligned}&\frac{\partial N}{\partial t}+u \frac{\partial N}{\partial x}+w \frac{\partial N}{\partial y}+\frac{bW_c}{C_\infty }\frac{\partial N}{\partial y}\left( \frac{\partial C}{\partial y}\right) =D_n\frac{\partial ^2 N}{\partial y^2}. \end{aligned}$$

The boundary conditions are as in^29,30^

12 $$\begin{aligned}&t<0 : u(x,y,t)=0, v(x,y,t)=0, w(x,y,t)=0, T(x,y,t)=T_\infty , C(x,y,t)=C_\infty , N (x,y,t)=N _\infty , \end{aligned}$$

13 $$\begin{aligned}&t\ge {0}: u(x,0,t)=U, v(x,0,t)=\Omega {x}, w(x,0,t)=0, -k_f\frac{\partial T}{\partial y}=h_f(T_f-T), D_B\frac{\partial C}{\partial y}+\frac{D_T}{T_\infty }\frac{\partial T}{\partial y}=0,\nonumber \\&\quad N(x,0,t)=N _w, u(x,\infty ,t)\rightarrow {U},v(x,\infty ,t)\rightarrow {0}, T(x,\infty ,t)\rightarrow {T_\infty }, C(x,\infty ,t)\rightarrow {C_\infty }, N (x,\infty ,t)\rightarrow {N _\infty }, \end{aligned}$$

where $\alpha _{1}$(>0) is the material parameter and ${u}_{{w}}$ is the stretching velocity.

Using the following transformations as in^29,30^ $\zeta =\sqrt{\frac{2c_{1}}{\nu _f}}\eta ^{\frac{1}{2}}y$, $\eta =1-e^{-c_{1}t}$, $u(x,y,t)=c_{1}xf^{\prime }(\eta ,\zeta )$, $v(x,y,t)=\Omega {g(\eta ,\zeta )}$, $w(x,y,t)=-\sqrt{2c_{1}\nu _f\eta }f^{\prime }(\eta ,\zeta )$, $\theta (\zeta )=\frac{T-T_\infty }{T_w-T_\infty }, \,\, \phi (\zeta )=\frac{C-C_\infty }{C_w-C_\infty }, \,\, \chi (\zeta )=\frac{N-N_\infty }{N_w-N_\infty }$, the above governing equations provide the following non-dimensional form

14 $$\begin{aligned}&f^{\prime \prime \prime }+\frac{1}{2}\zeta (1-\eta )f^{\prime \prime } -\eta \left( 1+\lambda _{1}{g^2}-{ff^{\prime ^2}}\right) +A_1\left( 2f^{\prime } f^{\prime \prime \prime }-f^{{\prime \prime }^2}\right) +\frac{A_2}{\sqrt{\eta }} f^{\prime \prime \prime \prime }g\nonumber \\&\quad -\frac{2\beta \theta }{(\zeta +d)^4}+\gamma _1\eta \left[ \theta -Nr\phi -Rb\chi \right] =0, \end{aligned}$$

15 $$\begin{aligned}&g^{\prime \prime }+\frac{1}{4}\zeta (1-\eta )g+\frac{1}{2}\eta (fg-f^{\prime }g) -2A_1f^{\prime \prime \prime }g=0, \end{aligned}$$

16 $$\begin{aligned}&\theta ^{\prime \prime }+Pr\left[ \frac{1}{4}\zeta (1-\eta )\theta ^{\prime } +\eta {f\theta ^\prime }+Nb\phi ^\prime \theta ^\prime +Nt\theta ^{\prime ^2}\right] +\eta \beta \lambda (\theta -\varepsilon )\frac{2{\Omega }g}{(\zeta +d)^3} +\eta {Pr}\beta \lambda (\theta -\varepsilon )\left[ \frac{2f^{\prime }}{(\zeta +d)^4}+\frac{4{\Omega }g}{(\zeta +d)^5}\right] =0, \end{aligned}$$

17 $$\begin{aligned}&\phi ^{\prime \prime }+Sc\left[ \frac{Nt}{Nb}\theta ^{\prime \prime }+\frac{1}{4} \zeta (1-\eta )\phi +\eta {f}\phi ^{\prime }\right] =0, \end{aligned}$$

18 $$\begin{aligned}&\chi ^{\prime \prime }-Pe\left[ \chi ^{\prime }\phi ^{\prime }+(\chi +N_\delta ) \phi ^{\prime \prime }\right] +\frac{Sb}{4}\zeta (1-\eta )\chi ^{\prime }+Sb\eta {f} \chi ^{\prime }=0, \end{aligned}$$

with non-dimensional boundary conditions given as

19 $$\begin{aligned}&f(\eta ,0)=f^{\prime}(\eta ,0)=0,\,\,\, g(\eta ,0)-1=0,\,\,\,\theta ^{\prime }=B_{i}(\theta -1),\,\,\, Nb\phi ^{\prime }(\eta ,0)+Nt\theta (\eta ,0)=0,\,\,\, \chi (\eta ,0)=1, \\&f^{\prime }(\eta ,\infty )\rightarrow {1},\,\,\, g(\eta ,\infty )\rightarrow {0},\,\,\, \theta (\eta ,\infty )\rightarrow {0},\,\,\, \phi (\eta ,\infty )\rightarrow {0},\,\,\, \chi (\eta ,\infty )\rightarrow {0}. \end{aligned}$$

Here ${c}_{1}$ is the constant such that ${c}_{1}$ > 0, $\eta$ designates the dimensionless time parameter, $\zeta$ indicates the converted variable, $f^{\prime }$ and *g* stand for the velocity components in the $x-$ and $y-$ directions, *T* means the dimensionless temperature, $\phi$ is the dimensionless nanoparticles concentration, *N* is density of motile microorganisms, $\gamma _1$ denotes the mixed convection parameter, *Nr* points out the buoyancy ratio, $\beta$ is the ferrohydrodynamic interaction parameter, *Pr* is the Prandtl number, *Sc* is the traditional Schmidt number, *Sb* is the bioconvection Schmidt number, $\lambda _1$ is the rotation parameter, $\lambda$ is the heat dissipation parameter, $\varepsilon$ is the curie temperature, *d* is the dimensionless distance, *Nt* is the thermophoresis, *Nb* is the Brownian motion parameter, $N_\delta$ denotes the microorganisms concentration difference parameter, *Rb* gives bioconvection Rayleigh number, *Pe* is the bioconvection Peclet number, *Re* is the Reynolds number, $A_1$ is the viscoelastic parameter, $A_2$ is the dimensionless parameter, *Bi* is the thermal Biot number and $\prime$ indicates the derivatives with respect to $\zeta$. These parameters are given in mathematical expressions by

$$\lambda _{1}=\left( \frac{\Omega }{c_{1}}\right) ^{2}$$, $$A_1=\frac{c_{1}\alpha _1}{\mu _{f_\infty }}$$, $$A_2=\frac{\alpha _1\Omega }{\mu _{f_\infty }}\sqrt{\frac{2c_1}{\nu _f}}$$, $$\beta =\frac{\gamma \mu _{0}K_{1}(T_\infty -T_w)\rho _{f_\infty }}{2\pi \mu _{f_\infty }^2}$$, $$d=\sqrt{\frac{2c_1a^2}{\nu _f}}$$, $$\gamma _1=\frac{\beta _Tg(1-C_\infty )(T_w-T_\infty )}{c_1^2R}$$, $$Nr=\frac{(\rho _p-\rho _{f_\infty })C_\infty }{\rho _{f_\infty }\beta _T(1-C_\infty )(T_w-T_\infty )}$$, $$Rb=\frac{(\rho _m-\rho _{f_\infty })\gamma ^{*}(N_w-N_\infty )}{\rho _{f_\infty }\beta _T(1-C_\infty )(T_w-T_\infty )}$$, $$Pr=\frac{\nu _f({\rho }c_p)_{f_\infty }}{k_f}$$, $$Nb=\frac{\tau {D_BC_\infty }}{\nu _f}$$, $$Nt=\frac{{D_T(T_w-T_\infty )}}{T_\infty \nu _f}$$, $$\lambda =\frac{c_1\mu ^{2}_{f_\infty }}{\rho _{f_\infty }(T_w-T_\infty )k_T}$$, $$\varepsilon =\frac{T_\infty }{T_\infty -T_w}$$, $$Sc=\frac{\nu _f}{D_B}$$, $$Sb=\frac{\nu _f}{D_B}$$, $$Pe=\frac{bW_c}{D_n}$$ and $${Bi}=\frac{h_f}{k_f}\left( \frac{\eta \nu _f}{2c_1}\right) ^{\frac{1}{2}}$$.

According to the dimensionless variables, the significant designed physical quantities termed as the skin friction factor $C_f$ , Nusselt number *Nu*, and the density of the motile microorganisms number *Nn* are given in the form

20 $$\begin{aligned} C_f=\frac{\tau _w}{\rho _{f_\infty }U^2},\,\,\,\,Nu= \frac{xq_r}{k_f(T_w-T_\infty )},\,\,\,\,Nn=\frac{xq_n}{D_n(\chi _w -\chi _\infty )}, \end{aligned}$$

where shear stress, surface heat and motile surface microorganisms fluxes are mathematically expressed as

21 $$\begin{aligned}&\tau _w=\mu _{f_\infty }\frac{\partial u}{\partial y}|_{y=0},\,\,\,\,q_r=-k_f\frac{\partial u}{\partial y}|_{y=0},\,\,\,\, q_n=-D_n\frac{\partial u}{\partial y}|_{y=0}. \end{aligned}$$

Using the similarity transformations and Eq. (21) through Eq. (20), the simplified forms are

22 $$\begin{aligned}&{Re^{\frac{1}{2}}}{{\eta}^{\frac{1}{2}}}C_f=2{\sqrt{2}}f^{\prime \prime }(\eta ,0), \\& {Re^{-\frac{1}{2}}}{\eta ^{\frac{1}{2}}}Nu=-{\sqrt{2}}\theta ^{\prime }(\eta ,0), \\&{Re^{-\frac{1}{2}}}{{\eta}^{\frac{1}{2}}}Nn=-{\sqrt{2}}N^{\prime }(\eta ,0), \end{aligned}$$

where $Re_{x}=\frac{cx^2}{\nu _f}$ is the Reynolds number.

## Solution by homotopy analysis method

For nonlinear systems of partial or ordinary differential equations, Homotopy Analysis Method (HAM) is recognized as an important alternative to the conventional numerical methods. Liao^67–69^ proposed the Homotopy Analysis Method, which uses the basic concepts of homotopy in topology to develop an alternative and general analytical-numerical method for nonlinear problems. The validity of HAM is independent of whether or not the considered equation contains small parameter(s). As a result, HAM overcomes the limitations of perturbation methods.

Taking the initial guesses and the linear operators as

23 $$\begin{aligned} f_{0}(\zeta )=A\zeta +(1-A)(1-e^{-\zeta }),\,\,g_{0}(\zeta )=e^{-\zeta },\,\, \theta _{0}(\zeta )=\frac{Bi}{1+Bi}e^{-\zeta },\,\,\phi _{0}(\zeta )=e^{-\zeta },\,\, \chi _{0}(\zeta )=e^{-\zeta }. \end{aligned}$$

Equation (23) satisfies the linear operators properties as given below

24 $$\begin{aligned}&L_{f}(E_{1}+E_{2}e^{\zeta }+E_{3}e^{-\zeta })=0,\,\,\,L_{g}(E_{4}e^{\zeta } +E_{5}e^{-\zeta })=0,\,\, L_{\theta }(E_{6}e^{\zeta }+E_{7}e^{-\zeta })=0,\,\,\,\nonumber \\&L_{\phi }(E_{8}e^{\zeta }+E_{9}e^{-\zeta })=0,\,\,\,L_{\chi }(E_{10}e^{\zeta } +E_{11}e^{-\zeta })=0, \end{aligned}$$

where $E_{i}(i=1,\ldots ,11)$ indicates the arbitrary constants.

The corresponding zeroth order form of the problems are

25 $$\begin{aligned}&(1-q)L_{f}[f(\zeta ,q)-f_{0}(\zeta )]=qh_fN_f[f(\zeta ,q),g(\zeta ,q), \theta (\zeta ,q),\phi (\zeta ,q),\chi (\zeta ,q)],\nonumber \\&(1-q)L_{g}[g(\zeta ,q)-g_{0}(\zeta )]=qh_gN_g[g(\zeta ,q),f(\zeta ,q)],\nonumber \\&(1-q)L_{\theta }[\theta (\zeta ,q)-\theta _{0}(\zeta )]=qh_{\theta }N_{\theta } [\theta (\zeta ,q),f(\zeta ,q),g(\zeta ,q),\phi (\zeta ,q)],\nonumber \\&(1-q)L_{\phi }[\phi (\zeta ,q)-\phi _{0}(\zeta )]=qh_{\phi }N_{\phi } [\phi (\zeta ,q),\theta (\zeta ,q),f(\zeta ,q)],\nonumber \\&(1-q)L_{\chi }[\chi (\zeta ,q)-\chi _{0}(\zeta )]=qh_{\chi }N_{\chi } [\chi (\zeta ,q),\phi (\zeta ,q),f(\zeta ,q)], \end{aligned}$$

with the boundary conditions

26 $$\begin{aligned}&f(0,q)=0,f^{\prime }(0,q)=1,\,\,f^{\prime }(\infty ,q)=A,\,\,g^{\prime }(0,q)=1,\,\, g(\infty ;q)=0,\,\,\theta ^{\prime }(0,q)=-Bi(1-\theta (0,q)),\nonumber \\&\theta (\infty ,q)=0,\phi ^{\prime }(0,q)=1, \phi (\infty ,q)=0\,\,\chi ^{\prime }(0,q)=1,\,\, \chi (\infty ;q)=0. \end{aligned}$$

The non-linear operators are given by

27 $$\begin{aligned}&{\mathbf {N}}_{f}[f(\zeta ,q),g(\zeta ,q),\theta (\zeta ,q),\phi (\zeta ,q),\chi (\zeta ,q)] = \frac{\partial ^3 f(\zeta ,q)}{\partial \zeta ^3} + \frac{1}{2}\zeta (1-\eta )\frac{\partial ^2 f(\zeta ,q)}{\partial \zeta ^2}\nonumber \\&\quad -\eta \left( 1+\lambda _{1}{g^2(\zeta ,q)}-f\left( \frac{\partial f(\zeta ,q)}{\partial \zeta }\right) ^2\right) +A_1\left( 2\frac{\partial f(\zeta ,q)}{\partial \zeta }\frac{\partial ^3 f(\zeta ,q)}{\partial \zeta ^3}-\left( \frac{\partial ^2 f(\zeta ,q)}{\partial \zeta ^2}\right) ^2\right) \nonumber \\&\quad +\frac{A_2}{\sqrt{\eta }}\frac{\partial ^4 f(\zeta ,q)}{\partial \zeta ^4}g(\zeta ,q) -\frac{2\beta \theta (\zeta ,q)}{(\zeta +d)^4} +\gamma _1(\zeta ,q)\eta \left[ \theta (\zeta ,q)-Nr\phi (\zeta ,q)-Rb\chi (\zeta ,q)\right] ,\nonumber \\&{\mathbf {N}}_{g}[f(\zeta ,q),g(\zeta ,q)] = \frac{\partial ^2 g(\zeta ,q)}{\partial \zeta ^2}+\frac{1}{4}\zeta (1-\eta )g(\zeta ,q)+\frac{1}{2}\eta (f(\zeta ,q)g(\zeta ,q) -\frac{\partial f(\zeta ,q)}{\partial \zeta }g(\zeta ,q))\nonumber \\&\quad -2A_1\frac{\partial ^3 f(\zeta ,q)}{\partial \zeta ^3}g(\zeta ,q), \end{aligned}$$

28 $$\begin{aligned}&{\mathbf {N}}_{\theta }[\theta (\zeta ,q),\phi (\zeta ,q),f(\zeta ,q),g(\zeta ,q)] = \frac{\partial ^2 \theta (\zeta ,q)}{\partial \zeta ^2}\nonumber \\&\quad +Pr\left[ \frac{1}{4}\zeta (1-\eta )\frac{\partial \theta (\zeta ,q)}{\partial \zeta }+\eta {f\theta (\zeta ,q)\frac{\partial \theta (\zeta ,q)}{\partial \zeta }} +Nb\frac{\partial \theta (\zeta ,q)}{\partial \zeta }\frac{\partial \phi (\zeta ,q)}{\partial \zeta }+Nt\left( \frac{\partial \theta (\zeta ,q)}{\partial \zeta }\right) ^2\right] \nonumber \\&\quad +\eta \beta \lambda (\theta -\varepsilon )\frac{2{\Omega }g(\zeta ,q)}{(\zeta +d)^3}+ \eta {Pr}\beta \lambda (\theta (\zeta ,q)-\varepsilon )\left[ \frac{2}{(\zeta +d)^4} \frac{\partial f(\zeta ,q)}{\partial \zeta }+\frac{4{\Omega }g(\zeta ,q)}{(\zeta +d)^5}\right] , \end{aligned}$$

29 $$\begin{aligned}&{\mathbf {N}}_{\phi }[\phi (\zeta ,q),\theta (\zeta ,q),f(\zeta ,q)] =\frac{\partial ^2 \phi (\zeta ,q)}{\partial \zeta ^2} \end{aligned}$$

30 $$\begin{aligned}&{\mathbf {N}}_{\phi }[\phi (\zeta ,q),\theta (\zeta ,q),f(\zeta ,q)] =\frac{\partial ^2 \phi (\zeta ,q)}{\partial \zeta ^2} &{\mathbf {N}}_{\chi }[\chi (\zeta ,q),\phi (\zeta ,q),f(\zeta ,q)] =\frac{\partial ^2 \chi (\zeta ,q)}{\partial \zeta ^2} -Pe\left[ \frac{\partial \chi (\zeta ,q)}{\partial \zeta }\frac{\partial \phi (\zeta ,q)}{\partial \zeta }+(\chi (\zeta ,q)+N_\delta )\frac{\partial ^2 \phi (\zeta ,q)}{\partial \zeta ^2}\right] \nonumber +\frac{Sb}{4}\zeta (1-\eta )\frac{\partial \chi (\zeta ,q)}{\partial \zeta }+Sb\eta {f(\zeta ,q)}\frac{\partial \chi (\zeta ,q)}{\partial \zeta }, \end{aligned}$$

where $q\in [0,1]$ is the embedding parameter. ${{\mathbf {N}}_{f}}$, ${{\mathbf {N}}_{f}}$, ${{\mathbf {N}}_{\theta }}$, ${{\mathbf {N}}_{\phi }}$ and ${\mathbf {N}}_{\chi }$ are the nonlinear operators.

The *m*th-order deformation problems are

31 $$\begin{aligned}&{L}_{f}[f_{m}(\zeta ,q)-{\xi _{m}}f_{m-1}(\zeta )]=h_{f}{\mathcal {R}}_{f,m}(\zeta ), \end{aligned}$$

32 $$\begin{aligned}&{L}_{g}[g_{m}(\zeta ,q)-{\xi _{m}}g_{m-1}(\zeta )]=h_{g}{\mathcal {R}}_{g,m}(\zeta ), \end{aligned}$$

33 $$\begin{aligned}&{L}_{\theta }[\theta _{m}(\zeta ,q)-{\xi _{m}}\theta _{m-1}(\zeta )] =h_{\theta }{\mathcal {R}}_{\theta ,m}(\zeta ), \end{aligned}$$

34 $$\begin{aligned}&{\mathcal {L}}_{\phi }[\phi _{m}(\zeta ,q)-{\xi _{m}}\phi _{m-1}(\zeta )] =h_{\phi }{\mathcal {R}}_{\phi ,m}(\zeta ), \end{aligned}$$

35 $$\begin{aligned}&{L}_{\chi }[\chi _{m}(\zeta ,q)-{\xi _{m}}\chi _{m-1}(\zeta )]=h_{\chi } {\mathcal {R}}_{\chi ,m}(\zeta ), \end{aligned}$$

with boundary conditions

36 $$\begin{aligned}&f_{m}(0)=f^{\prime }_{m}(0)=f^{\prime }_{m}(\infty )=0,\nonumber \\&g^{\prime }_{m}(0)=g_{m}(0)=g_{m}(\infty )=0,\nonumber \\&\theta ^{\prime }_{m}(0)-B_{i1}\theta _{m}(0)=\theta _{m}(\infty )=0,\nonumber \\&\phi ^{\prime }_{m}(0)=\phi _{m}(0)=\phi _{m}(\infty )=0,\nonumber \\&\chi ^{\prime }_{m}(0)=\chi _{m}(0)=\chi _{m}(\infty )=0, \end{aligned}$$

where

37 $$\begin{aligned} \xi _{m}= {\left\{ \begin{array}{ll} 0,\,\, \text {if }m\le {1}\\ 1,\,\, \text {if }m>1. \end{array}\right. } \end{aligned}$$

And

38 $$\begin{aligned} {\mathcal {R}}_{f}^{m}(\zeta )&={f'''_{m-1}}+\frac{1}{2}\zeta (1-\eta ) f^{\prime \prime }_{m-1}-\eta \left( 1+\lambda _{1}{g^2_{m-1}}-{\sum _{r=0}^{m-1} \left( \sum _{k=0}^{r}{f_{m-1-r}f'_{r-k}}\right) {f'_{k}}}\right) \nonumber \\&\quad +A_1\left( 2\sum _{r=0}^{m-1}{f'_{m-1-r}f'''_{r}}-\sum _{r=0}^{m-1}{f''_{m-1-r}f''_{r}} \right) +\frac{A_2}{\sqrt{\eta }}\sum _{r=0}^{m-1}{g_{m-1-r}f''''_{r}} -\frac{2\beta \theta _{m-1}}{(\zeta +d)^4}\nonumber \\&\quad +\gamma _1\eta \left[ \theta _{m-1}-Nr\phi _{m-1}-Rb\chi _{m-1}\right] =0, \end{aligned}$$

39 $$\begin{aligned} {\mathcal {R}}_{g}^{m}(\zeta )&=g^{\prime \prime }_{m-1}+\frac{1}{4}\zeta (1-\eta )g_{m-1} +\frac{1}{2}\eta \left( \left( \sum _{r=0}^{m-1}{f_{m-1-r}g_{r}}\right) - \sum _{r=0}^{m-1}\left( {f'_{m-1-r}g_{r}}\right) \right) \nonumber \\&\quad -2A_1\sum _{r=0}^{m-1}{f'''_{m-1-r}}{g_{r}}, \end{aligned}$$

40 $$\begin{aligned} {\mathcal {R}}_{\theta }^{m}(\zeta )&={\theta }''_{m-1}+Pr\left[ \frac{1}{4}\zeta (1- \eta )\theta ^{\prime }_{m-1}+\eta \sum _{r=0}^{m-1}{f_{m-1-r}}{\theta '_{r}} +Nb\sum _{r=0}^{m-1}{\phi '_{m-1-r}}{\theta '_{r}}+Nt\sum _{r=0}^{m-1} {\theta '_{m-1-r}}{\theta '_{r}}\right] \nonumber \\&\quad +\eta \beta \lambda \frac{2{\Omega }\sum _{r=0}^{m-1}{g_{m-1-r}}{\theta _{r}}}{(\zeta +d)^3}-\eta \beta \lambda \frac{2\varepsilon {\Omega }g_{m-1}}{(\zeta +d)^3}+ \eta {Pr}\beta \lambda \left[ \frac{2\sum _{r=0}^{m-1}{f'_{m-1-r}}{\theta _{r}}}{(\zeta +d)^4}+\frac{4{\Omega }\sum _{r=0}^{m-1}{g_{m-1-r}}{\theta _{r}}}{(\zeta +d)^5}\right] \nonumber \\&\quad -\eta {Pr}\beta \lambda \varepsilon \left[ \frac{2f^{\prime }_{m-1}}{(\zeta +d)^4} +\frac{4{\Omega }g_{m-1}}{(\zeta +d)^5}\right] , \end{aligned}$$

41 $$\begin{aligned} {\mathcal {R}}_{\phi }^{m}(\zeta )&={\phi ''_{m-1}}+Sc\left[ \frac{Nt}{Nb} \theta ^{\prime \prime }_{m-1}+\frac{1}{4}\zeta (1-\eta )\phi _{m-1} +\eta \sum _{r=0}^{m-1}{f_{m-1-r}}{\phi '_{r}}\right] , \end{aligned}$$

43 $$\begin{aligned} {\mathcal {R}}_{\chi }^{m}(\zeta )&=\chi ''_{m-1}+{Pe}\left[ \sum _{r=0}^{m-1} \phi '_{m-1-r}\chi '_{r}+\sum _{r=0}^{m-1}\phi ''_{m-1-r}\chi _{r}+N_{\delta } \phi ''_{m-1}\right] + \frac{Sb}{4}\zeta (1-\eta )\chi ^{\prime }_{m-1}\nonumber \\&\quad +Sb\eta \sum _{r=0}^{m-1}f_{m-1-r}\chi '_{r}. \end{aligned}$$

The general solution is given by

44 $$\begin{aligned} f_m(\zeta )&=f_{m}^{*}(\zeta )+E_{1}+E_{2}e^\zeta +E_{3}e^{-\zeta }, \end{aligned}$$

45 $$\begin{aligned} g_m(\zeta )&=g_{m}^{*}(\zeta )+E_{4}e^\zeta +E_{5}e^{-\zeta }, \end{aligned}$$

46 $$\begin{aligned} \theta _m(\zeta )&=\theta _{m}^{*}(\zeta )+E_{6}e^\zeta +E_{7}e^{-\zeta }, \end{aligned}$$

47 $$\begin{aligned} \phi _m(\zeta )&=\phi _{m}^{*}(\zeta )+E_{8}e^\zeta +E_{9}e^{-\zeta }, \end{aligned}$$

48 $$\begin{aligned} \chi _m(\zeta )&=\chi _{m}^{*}(\zeta )+E_{10}e^\zeta +E_{11}e^{-\zeta }, \end{aligned}$$

where $f_{m}^{*}(\zeta ),(g_{m}^{*}(\zeta ),\theta _{m}^{*}(\zeta ),\phi _{m}^{*}(\zeta )$ and $\chi _{m}^{*}(\zeta )$ are the special solutions.

## Results and discussion

Solution authentication has an important role in the evaluations of the problems. Therefore, the present solution is compared with the published work. Order of approximation of the present work in Table 1 is given which presents the close agreement with the published paper results^28^.

**Table 1:** Comparison of the current work.

Order of approximation	$\hbox {f}^{\prime \prime }$(0)^28^	$\hbox {f}^{\prime \prime }$(0) (Present)	$\hbox {g}^{\prime }$(0)^28^	$\hbox {g}^{\prime }$(0) (Present)
1	1.59936137	1.59936125	0.20487518	0.20487515
2	1.59936085	1.59936081	0.204875662	0.204875661
3	1.59936095	1.59936095	0.204875662	0.204875662
15	1.59936095	1.59936095	0.204875662	0.204875662

### Velocity profiles

**Figure 2:**
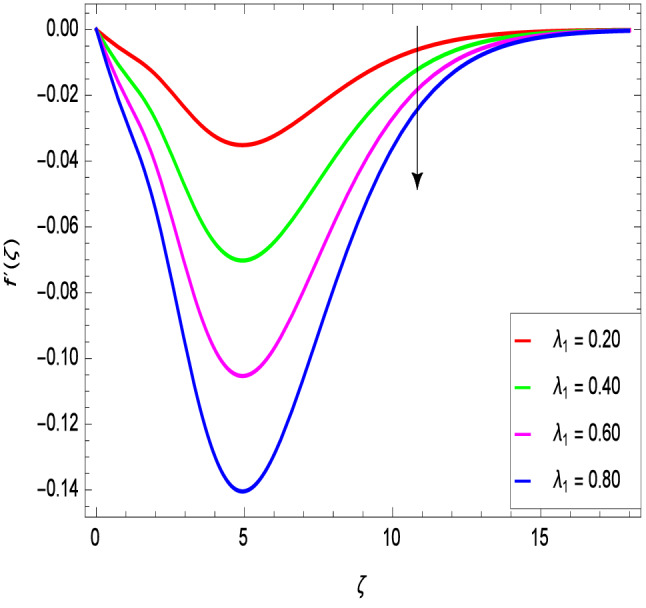
$f^{\prime }(\zeta )$ in terms of $\lambda _1$.

**Figure 3:**
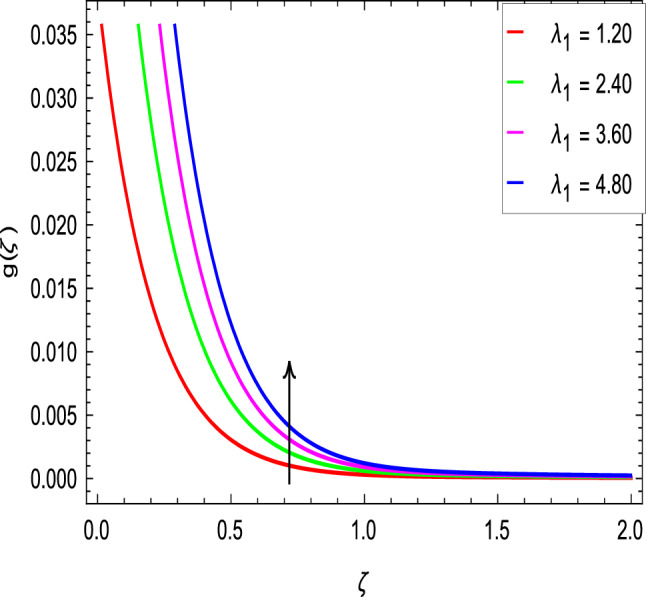
$g(\zeta )$ in terms of $\lambda _1$.

**Figure 4:**
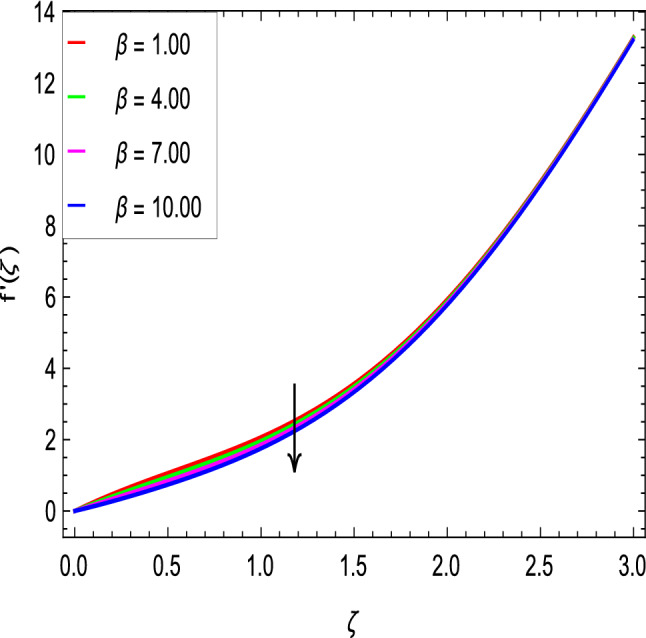
$f^{\prime }(\zeta )$ in terms of $\beta$.

**Figure 5:**
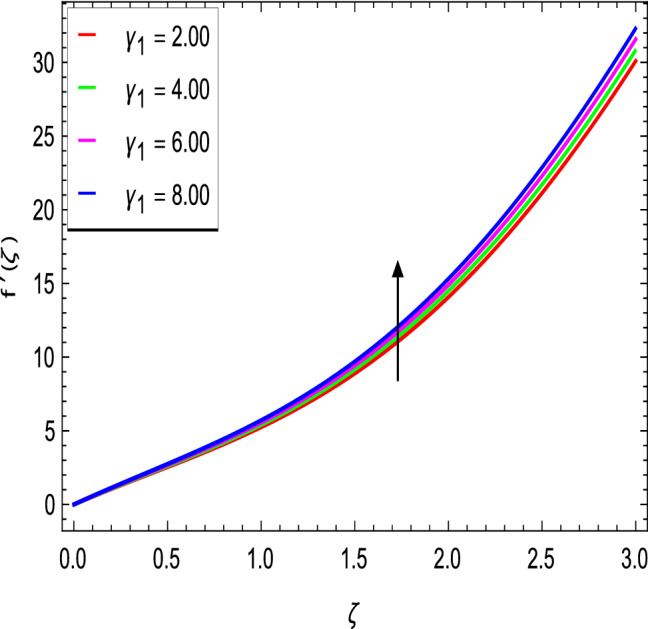
$f^{\prime }(\zeta )$ in terms of $\gamma _1$.

**Figure 6:**
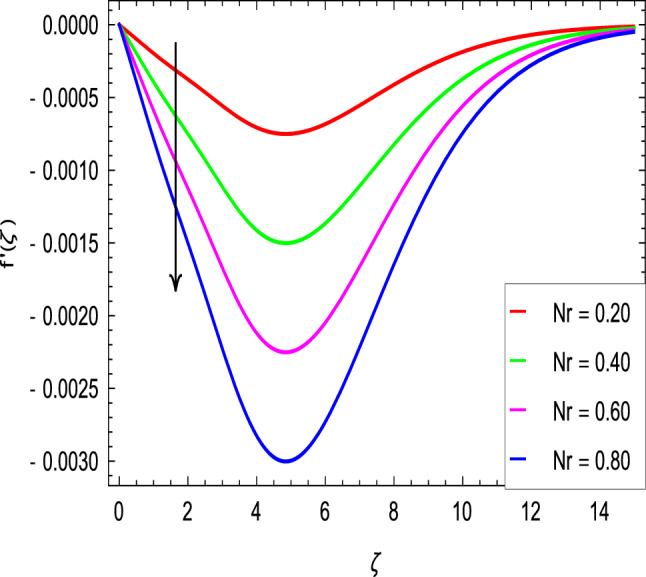
$f^{\prime }(\zeta )$ in terms of *Nr*.

**Figure 7:**
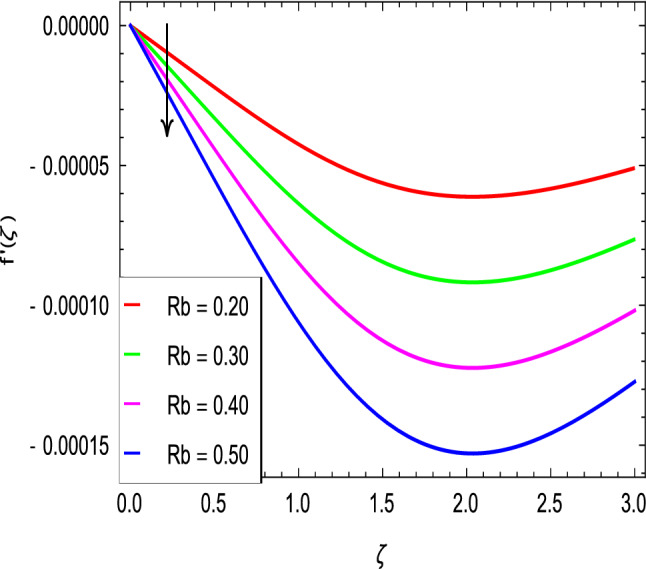
$f^{\prime }(\zeta )$ in terms of *Rb*.

**Figure 8:**
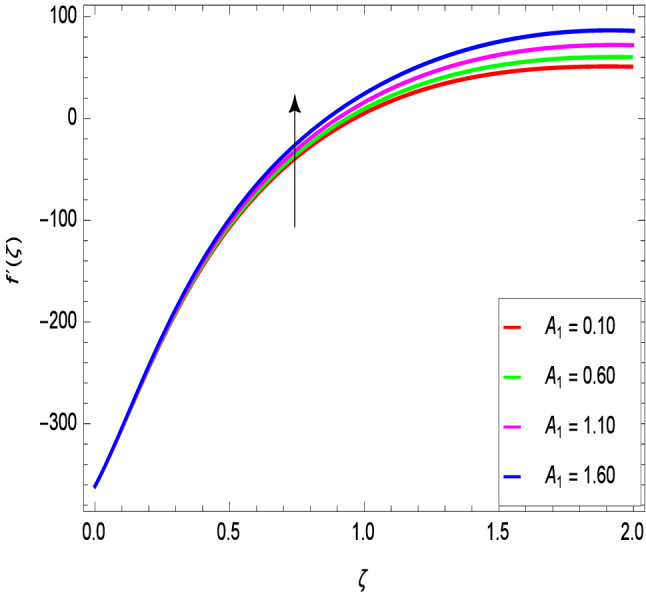
$f^{\prime }(\zeta )$ in terms of $A_1$.

**Figure 9:**
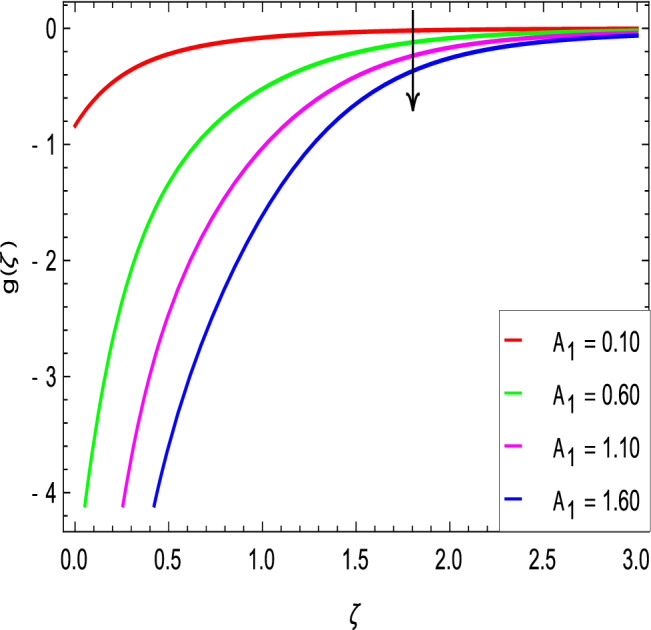
$g(\zeta )$ in terms of $A_1$.

Figures 2 and 3 show the impact of the rotation parameter on the velocity profiles. For elevating values of the rotation parameter $\lambda _1$, the velocity $f^\prime$ is weakened and the velocity *g* is enhanced by higher values of rotation parameter $\lambda _1$. The physical interpretation for this feature is attributed to the reduction of momentum and thermal boundary layers, which lead to an increase in the gradients of nanofluid velocity. Figure 4 shows a declining trend in velocity $f^\prime$ for the higher estimation of the ferromagnetic parameter $\beta$. Physically higher values develop more resistance to fluid flow. At the end, it reduces the velocity profile. Figure 5 shows a growing trend in velocity $f^\prime$ for the larger values of $\gamma _1$. This can be perceived as buoyancy aspects understanding, the convection cooling effects are enhanced by a strong acceleration of the flow. Figure 6 shows that the dimensionless velocity decreases with an increase in the buoyancy ratio parameter *Nr* leading to an increase in the negative buoyant force caused by the presence of nanoparticles. The effect of increasing the bioconvection Rayleigh number *Rb* is that the convection power caused by bioconvection is enhanced against the convection of the buoyancy force. As a result, it could be noted that the flow velocity decreases with increasing the bioconvection Rayleigh number *Rb* values as shown in Fig. 7. Figure 8 is plotted here to measure the velocity variance against the viscoelastic parameter $A_1$. The increasing velocity trend is aligned with the rising viscoelastic parameter $A_1$. This behavior is rationalized by the mathematical representation of $A_1 = \alpha _1c_1/\mu _{f_\infty }$ that, by increasing the magnitude of $A_1$, the viscosity decreases due to the velocity of the fluid. Mounts and the chaotic behavior of the uplifters are seen. It is efficient to note that the present problem reduces to the Newtonian case when $A_1 = 0$. From the boundary layer point of view, the thickness of the fluid increases with an increase in the viscoelastic parameter $A_1$. The opposite trend of velocity *g* for the viscoelastic parameter $A_1$ is observed as shown in Fig. 9.

### Temperature profile

**Figure 10:**
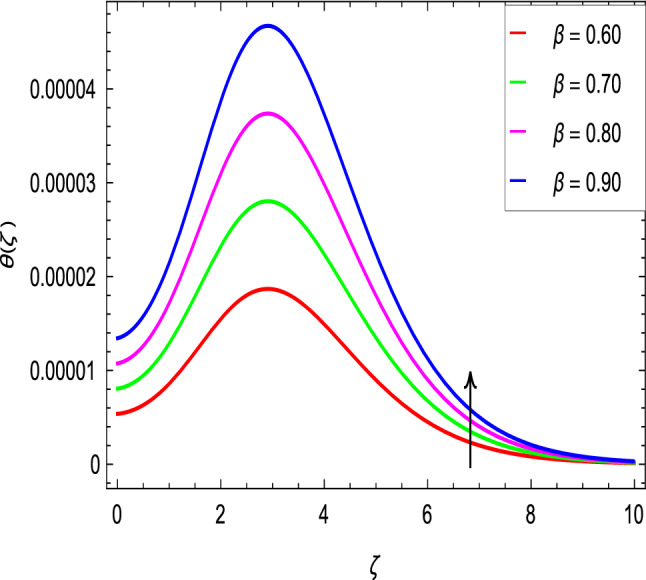
$\theta (\zeta )$ in terms of $\beta$.

**Figure 11:**
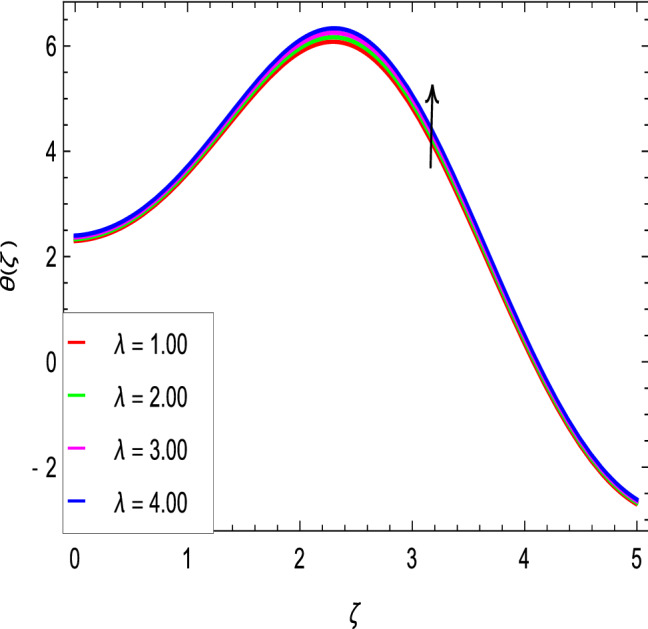
$\theta (\zeta )$ in terms of $\lambda$.

**Figure 12:**
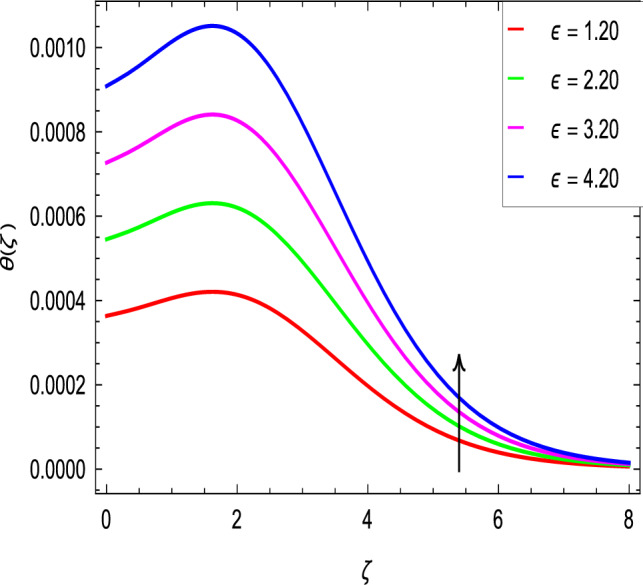
$\theta (\zeta )$ in terms of $\varepsilon$.

**Figure 13:**
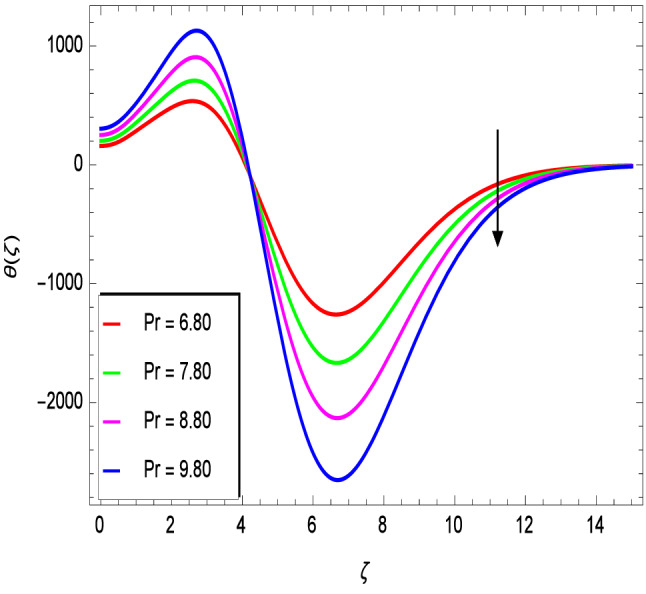
$\theta (\zeta )$ in terms of *Pr*.

**Figure 14:**
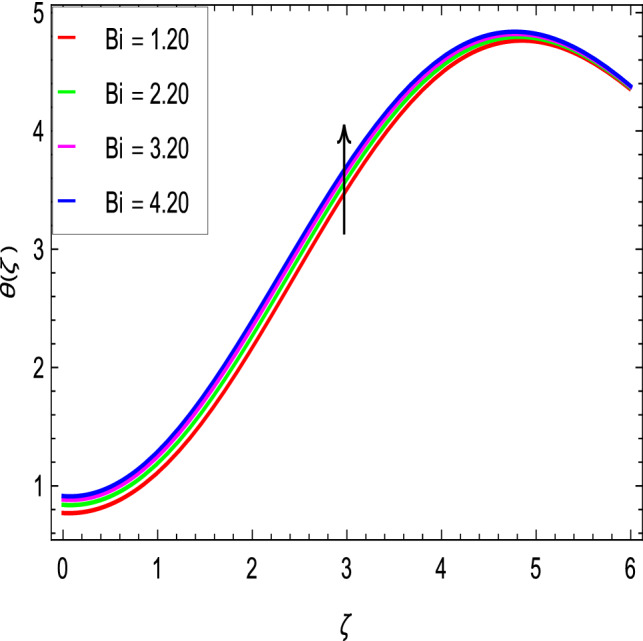
$\theta (\zeta )$ in terms of *Bi*.

**Figure 15:**
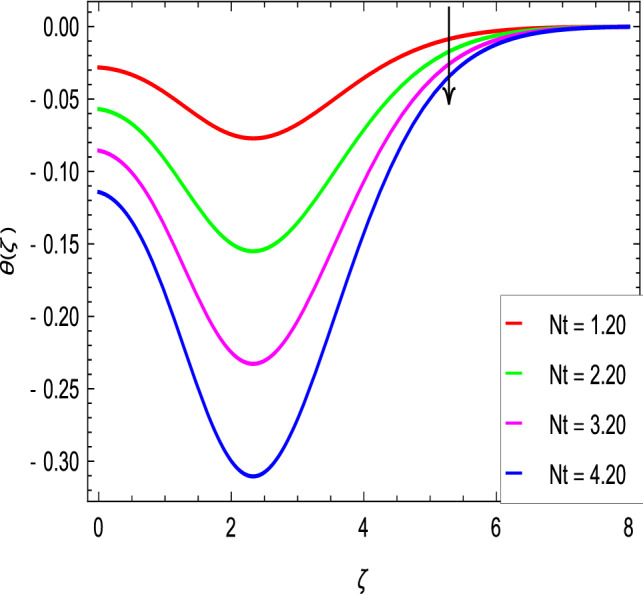
$\theta (\zeta )$ in terms of *Nt*.

**Figure 16:**
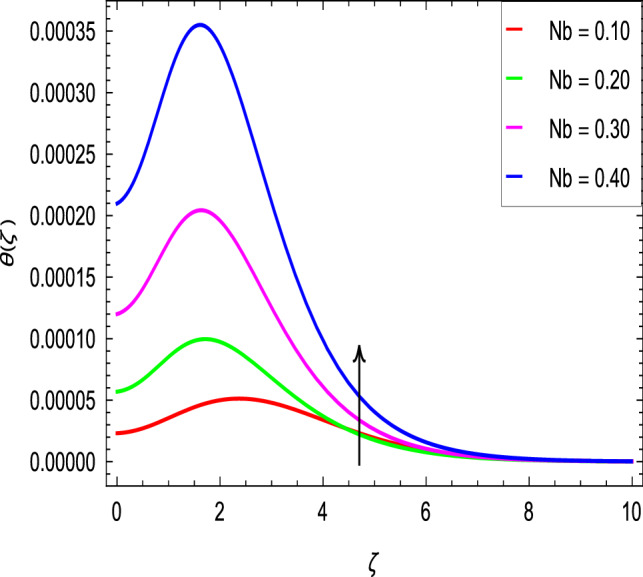
$\theta (\zeta )$ in terms of *Nb*.

Figure 10 is used to measure the effect of the ferromagnetic parameter $\beta$ on temperature. The temperature here rises with the greater values of ferromagnetic parameter $\beta$. The effect of the heat dissipation parameter $\lambda$ on temperature is shown in Fig. 11. The temperature here increases with an increase in the the heat dissipation parameter values. Physically thermal conductivity of the fluid decays with the higher values of heat dissipation parameter $\lambda$. The temperature characteristics with the curie temperature parameter $\varepsilon$ are shown in Fig. 12. The temperature decreases in the estimation of the curie temperature parameter $\varepsilon$. Nanofluid thermal conductivity increases with the increase in the curie temperature $\varepsilon$. In effect, the added heat is removed and the temperature rises from the surface to the nanofluid. Figure 13 shows variations in temperature for the increasing values of Prandtl number *Pr*. For the higher values of the Prandtl number *Pr*, the temperature of the nanofluid decreases. Figure 14 shows the response of the thermal Biot number *Bi* to the temperature profile. The temperature is indicated to rise as a result of the increase in the thermal Biot number values. Thermal Biot number *Bi* depends on the coefficient of heat transfer or is directly proportional to the coefficient of heat transfer. Figure 15 shows that the non-dimensional temperature and thermal boundary layer thickness decrease as the thermophoresis parameter *Nt* increases. The extra energy produced by the interaction of nanoparticles with the fluid due to the thermophoresis effect reduces the temperature. As a result, the thickness of the thermal boundary layer is reduced by higher values of the thermophoresis parameter *Nt*. As shown in Fig. 16, the dimensionless temperature and thermal boundary layer thickness are increased with an increase in Brownian motion parameter *Nb*. The additional heat generated by the interaction between nanoparticles and the fluid due to Brownian motion increases the temperature. As a result, the thermal boundary layer thickness enhances with the positive values of the Brownian motion parameter *Nb*.

### Nanoparticles concentration profile

**Figure 17:**
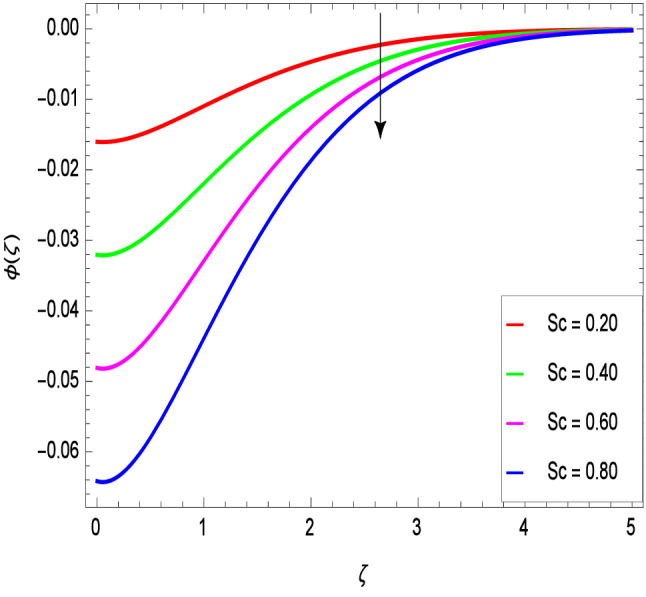
$\phi (\zeta )$ in terms of *Sc*.

**Figure 18:**
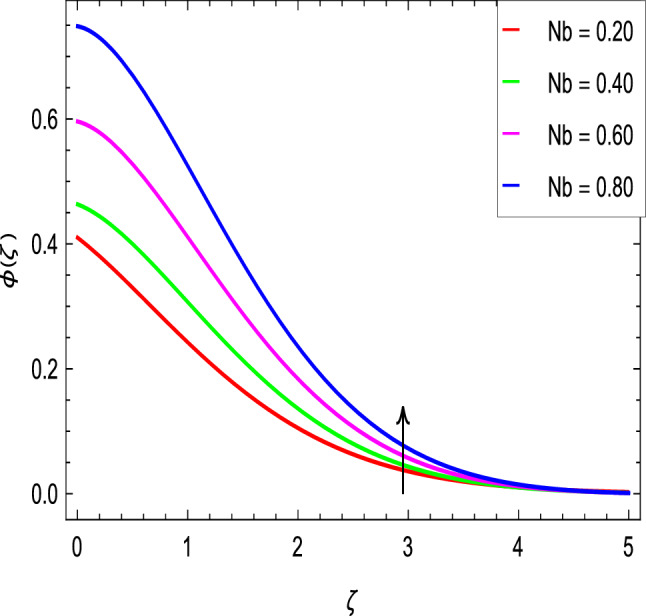
$\phi (\zeta )$ in terms of *Nb*.

**Figure 19:**
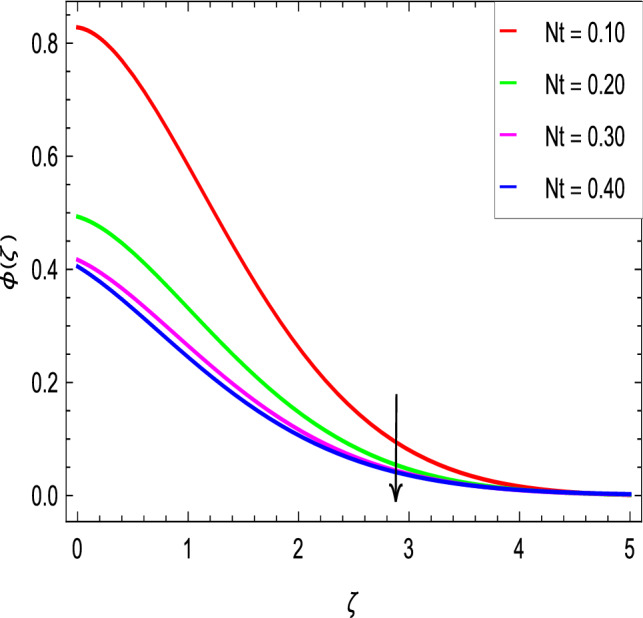
$\phi (\zeta )$ in terms of *Nt*.

The Schmidt number *Sc* is attributed to mass diffusion and therefore increases the mass diffusivity leading to a lower concentration of nanoparticles due to less mass diffusion transport, as shown in Fig. 17. Figure 18 shows that the concentration of nanoparticles and hence the thickness of concentration boundary layer increase with the increase of the Brownian motion parameter *Nb*. Figure 19 shows that the concentration of nanoparticles decreases as thermophoresis parameter *Nt* increases. Thus, it can be deduced that the boundary layer thickness of nanoparticles with the thermophoresis parameter *Nt* becomes less thick.

### Motile microoganisms concentration profile

**Figure 20:**
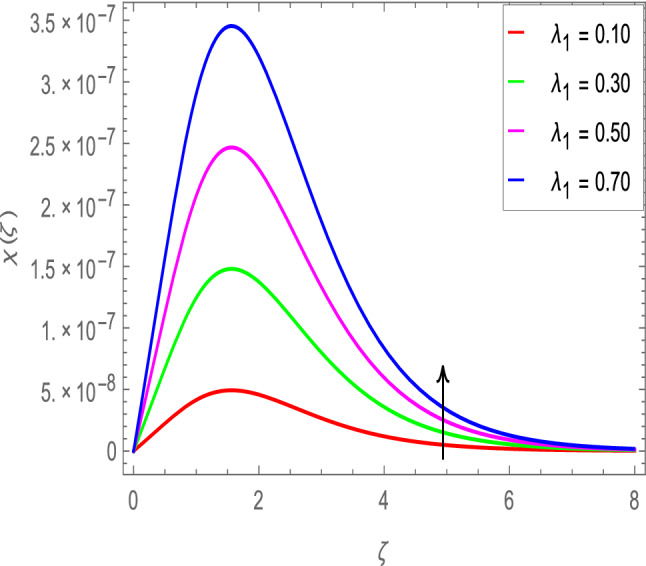
$\chi (\zeta )$ in terms of $\lambda _1$.

**Figure 21:**
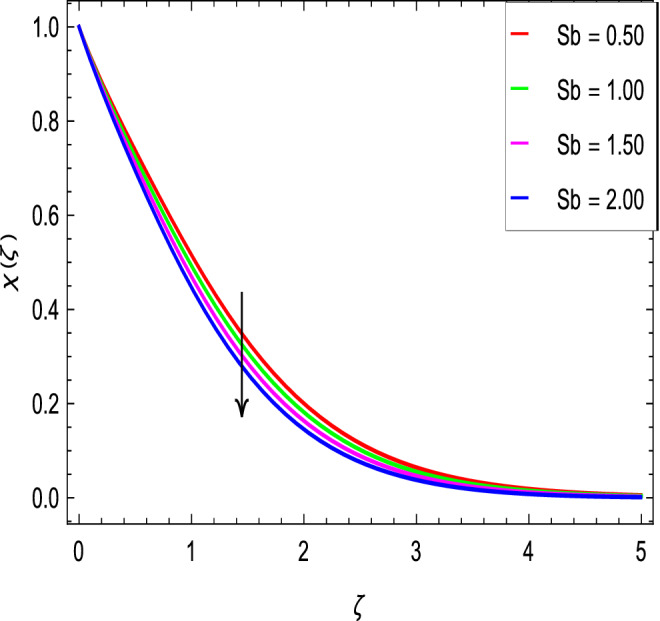
$\phi (\zeta )$ in terms of *Sb*.

**Figure 22:**
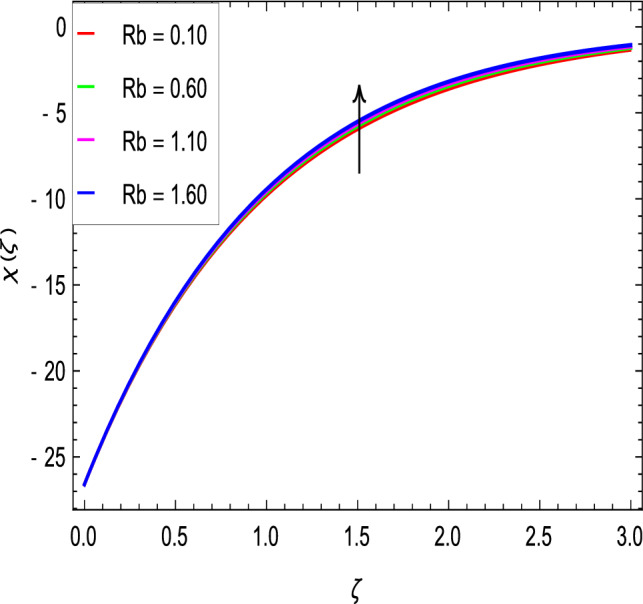
$\chi (\zeta )$ in terms of *Rb*.

**Figure 23:**
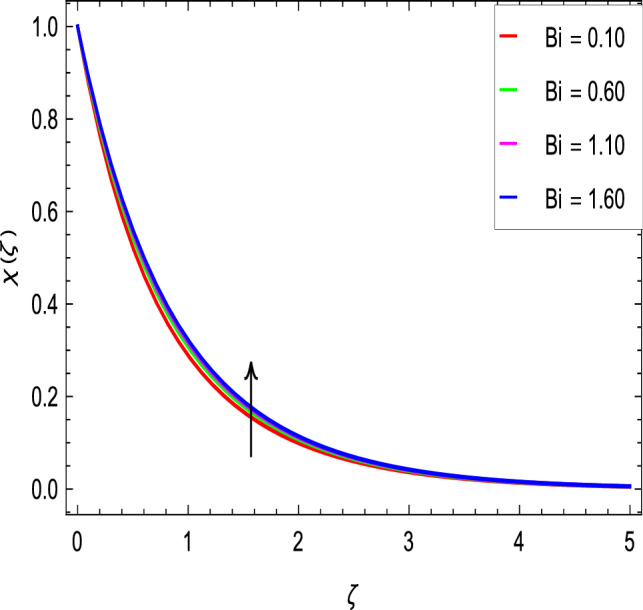
$\chi (\zeta )$ in terms of *Bi*.

**Figure 24:**
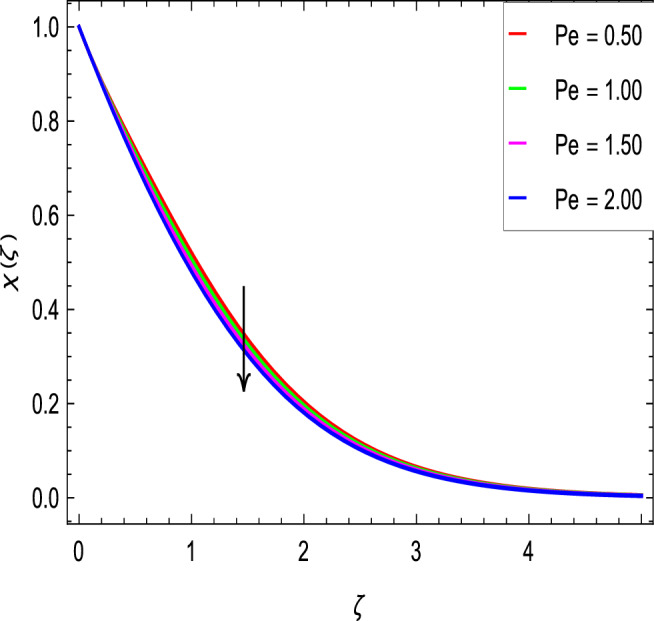
$\chi (\zeta )$ in terms of *Pe*.

**Figure 25:**
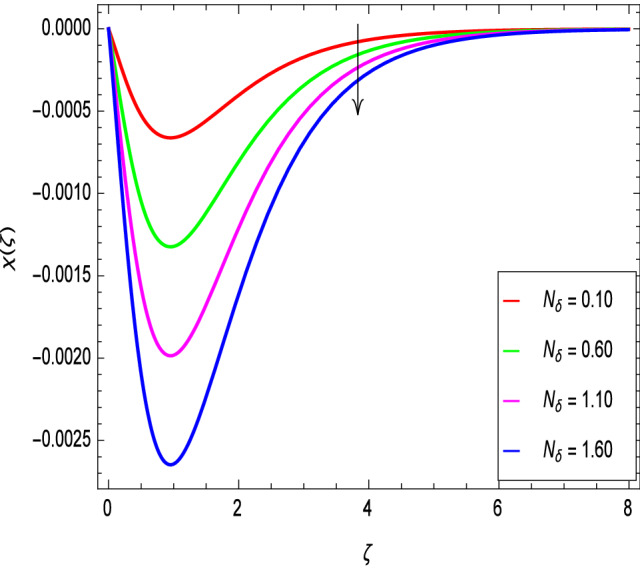
$\chi (\zeta )$ in terms of $N_\delta$.

Figure 20 shows the effect of the rotation parameter $\lambda _{1}$ on the motile microorganism density for the elevated values which increases the density of the motile microorganisms. Due to the strong relation of parameter in the governing equation 18 for the microorganisms, the dimensionless density of the motile microorganisms is highly influenced by the bioconvection Schmidt number *Sb*. It can be seen from Fig. 21 that the rising values of *Sb* reduces the dimensionless density of the motile microorganisms profile. This is due to the bioconvection Schmidt number *Sb* which aids in the weakening of microorganisms concentration layer thickness, as indicated. The effect of the bioconvection Rayleigh number *Rb* on the density of motile microorganisms fluctuations is shown in Fig. 22. It is clear that the bioconvection Rayleigh number *Rb* is enhancing the motile microorganisms. Of course, the Biot number *Bi* strengthens the convective heat transfer from the hot nanofluid domain to the cold nanofluid portion on the surface of the sphere. It is clear that the improvement in the Biot number *Bi* results in more improvement in convective heating and density of motile microorganisms increases as seen in Fig. 23. The effects of Peclet number *Pe* and the concentration difference parameter on the motile microorganisms profile are shown in Figs. 24 and 25 respectively. This would be related to the fact that the concentration of motile micro-organisms within the boundary layer decreases as these parameters increase.

## Conclusion

Analysis of the themodynamics with the dipole effect and microorganisms shows that flow and heat transfer are enhanced with rotation parameter while for the magnetic dipole effect they have the opposite trend. Prandtl number has the cooling effect and the heat transfer increases with convective conditions. Nanoparticles concentration is improved with the Brownian motion parameter while the motile microorganisms motion is also enhanced with the rotation parameter, bioconvection Rayleigh number and convective condition.

## Data Availability

Availability exists for whole of the data.
